# Supplementary material for: Enhancement of Sphingomyelinase-Induced Endothelial Nitric Oxide Synthase-Mediated Vasorelaxation in a Murine Model of Type 2 Diabetes
Source: Int J Mol Sci. 2023 May 6;24(9):8375. doi: 10.3390/ijms24098375 (PMC10179569; doi:10.3390/ijms24098375)
Supplement: Supplementary file 1 [file ijms-24-08375-s001.zip › ijms-2284055-supplementary.pdf]

Figure S1.

A

control	db/db
24.8	43.6
23.8	49.2
25.8	38.4
26.4	46.3
34	51.2
24.7	47.3
35.7	38.9
31.9	51.4
26	49.3
26.3	55.3
24.7	52
24.6	55.8
36.4	63.6
28.4	67
24	67
27	65
23.4	64.2
33	53.9
32.5	52.3
30.1	48.3
26.4	39.9
30.7	27.3

B

control	db/db
15.1	21.6
12.4	40.8
10.4	25.8
15.5	43.8
9.3	33.8
11	43
11	40
11.5	19.7
14.1	33
11.8	22.8
10.1	30.1
13.3	16.4
13.2	26.6
6.5	26.6
10.5	21.8
10.1	28.8
11.4	36.6
8.8	25.6
7.7	28.4
9.3	29
8.4	54.6

C

control	db/db
44.66	43.24
8.86	80.48
18.89	77.61
14.59	36.07
17.45	93.37
51.83	90.51
33.21	137.77
4.12	36.07
1.42	17.07
14.55	1.42
31.29	182.49
1.42	19.77
1.42	82.21
	23.61

D

control						db/db				
Ach -9	Ach -8	Ach -7	Ach -6	Ach -5		Ach -9	Ach -8	Ach -7	Ach -6	Ach -5
1.81	18.41	82.29	86.47	86.89		0.78	7.20	49.81	53.52	53.52
3.08	14.84	70.13	70.13	71.87		3.23	17.96	39.56	39.56	39.56
14.11	42.47	77.53	77.53	77.53		0.00	3.61	25.10	41.29	41.29
1.40	21.66	57.47	64.49	64.49		0.00	6.47	45.96	45.96	45.96
1.68	3.53	51.68	62.52	62.52		0.00	11.30	66.28	75.37	75.37
1.15	1.15	6.76	42.89	42.89		0.00	2.41	20.13	20.13	20.13
9.16	17.88	46.78	64.23	64.23		0.73	0.73	72.38	75.63	75.63
0.00	33.06	76.63	76.94	83.78		1.38	1.76	80.20	80.20	80.20
5.33	12.92	74.50	75.75	80.33		0.00	3.55	17.27	52.49	56.09
0.00	0.00	12.37	27.34	27.34		0.00	0.00	13.51	44.59	46.39
0.00	4.13	32.26	57.68	57.68		0.00	2.34	33.64	55.65	55.65
0.00	0.00	29.35	68.75	68.75		0.00	0.00	26.12	36.85	36.85
						0.00	0.46	52.41	57.03	57.03
						0.00	19.51	41.09	50.55	50.55
						2.95	2.95	32.15	49.97	49.97
						2.73	2.73	22.70	49.20	49.20
						0.00	0.00	26.81	34.27	34.27
						0.00	0.00	9.36	32.32	32.32
						0.00	3.52	44.94	67.74	67.74
						0.00	0.00	38.14	74.26	74.26
						10.26	10.26	43.36	59.32	67.48
						3.64	3.64	32.18	39.95	43.76
						0.00	0.00	16.67	29.87	33.02
						0.00	0.00	23.20	30.26	40.10

E

control							db/db					
SNP -10	SNP -9	SNP -8	SNP -7	SNP -6	SNP -5		SNP -10	SNP -9	SNP -8	SNP -7	SNP -6	SNP -5
1.19	5.95	53.21	87.38	100.83	104.40		0.00	0.00	60.94	98.78	106.97	113.43
2.73	8.77	44.44	81.29	96.88	102.14		0.00	0.00	65.35	92.74	99.35	102.23
12.94	24.47	63.06	89.65	110.59	104.00		0.00	3.54	40.36	83.60	95.74	99.94
6.99	12.50	45.04	78.68	96.69	103.31		0.00	0.00	39.27	81.84	96.93	100.60
0.00	0.00	51.03	82.34	99.73	104.37		0.00	0.00	48.34	87.70	97.48	101.70
0.00	0.00	52.39	79.57	90.12	96.38		0.00	0.00	8.95	79.10	92.50	98.55
4.49	16.47	58.38	92.22	102.40	103.29		0.00	0.00	33.01	95.10	109.58	114.25
2.59	2.59	60.00	107.41	112.82	113.18		0.00	0.00	58.20	94.77	102.08	108.45
1.66	12.81	51.96	100.47	112.34	112.34		0.00	2.12	32.90	91.21	99.09	106.97
2.00	8.09	63.27	96.67	107.99	110.09		0.00	0.00	21.16	92.86	105.81	112.13
0.00	0.00	55.56	96.00	108.00	112.74		0.00	0.00	15.54	80.58	100.14	108.76
0.00	0.00	36.05	77.36	103.30	106.60		0.00	0.00	56.11	92.53	103.55	107.04
							0.00	0.00	29.79	88.25	98.23	102.23
							0.00	0.00	53.79	83.45	95.86	97.93
							0.00	0.00	44.71	87.67	97.15	100.55
							0.00	0.00	34.01	84.61	97.30	102.39
							0.00	0.00	8.28	66.63	83.96	93.85

Figure S2.

A

Data table contains 51 + 49 columns (aorta samples from control and db/db mice) times 1200 rows (one registered data point every second for 20 minutes). Can be sent upon request from the corresponding author (benyo.zoltan@med.semmelweis-univ.hu).

B

control	db/db
76173.49	-12282.53
20960.91	-21343.18
-7237.95	-53502.12
8806.94	-21960.38
-16377.69	-15816.39
1387.52	-22671.05
-1924.60	6833.89
67600.51	6450.95
6889.60	-13653.05
28803.92	12017.17
18428.14	7817.29
-4868.91	48774.09
30225.56	-12947.30
29257.07	-34694.24
8557.75	-17831.47
-4787.73	-49123.05
-9692.02	-38554.00
-11434.69	-24872.85
1158.19	-25884.86
-10716.90	-56220.22
-33701.45	-15993.37
3067.16	1168.05
41664.31	6602.33
31150.52	-10340.74
5479.31	14380.50
16415.47	-1519.42
4055.17	-27508.79
37127.61	23040.57
46661.33	-8571.76
16808.98	-2605.97
51559.84	7126.86
14985.08	-25698.89
42325.89	8132.12
18718.03	-3254.83
12934.94	-32251.31

1905.85	-28788.81
4571.66	-31869.88
5586.14	29809.47
-3849.76	1418.87
18496.31	3209.14
-5513.20	14683.24
13521.71	26707.13
-5761.43	37520.84
30504.10	-14066.74
-23126.48	-3857.31
25148.97	14334.78
-3456.05	-20948.78
8251.47	-10953.56
28740.57	-20967.13
12796.88	
15741.58	

C

control	db/db
130.80	-48.35
116.45	-39.57
-33.81	-65.98
42.13	-66.42
-48.86	-62.62
35.61	-45.33
55.07	10.71
98.71	-30.34
39.05	-35.61
66.67	47.81
70.87	13.82
-24.22	69.46
45.58	-40.07
33.17	-79.20
29.18	-58.11
-13.89	-64.09
-30.32	-67.54
-31.69	-46.51
-22.13	-50.14
-20.59	-74.52
-43.22	-35.01
16.98	10.90

47.20	12.37
36.42	-29.89
11.15	27.65
20.62	-18.64
12.98	-36.83
43.90	41.46
56.64	-63.66
25.63	-33.82
74.53	22.49
27.00	-34.15
52.48	13.09
26.49	-58.52
19.73	-49.74
11.56	-74.92
13.99	-59.91
15.82	58.11
-15.35	-50.34
31.54	-44.81
-43.23	-31.54
19.22	58.19
-13.81	69.23
38.53	-30.64
-45.32	-58.10
36.73	26.08
-25.07	-74.52
37.17	-24.56
45.58	-77.74
19.30	
37.34	

Figure S3.

A

Data table contains 20 + 20 columns (aorta samples from control and db/db mice) times 1200 rows (one registered data point every second for 20 minutes). Can be sent upon request from the corresponding author (benyo.zoltan@med.semmelweis-univ.hu).

B

control	db/db
-34275.63	-33340.19
-46184.43	-22019.32

15773.53	-38984.76
8624.41	-18256.83
8303.24	-18819.10
-10476.81	-19506.19
3199.94	-30397.64
-6091.18	-17615.86
-6085.67	-13057.16
-26869.72	-23396.83
4309.81	-81987.19
744.68	-71412.78
8299.02	1390.10
12514.62	-53867.95
-593.11	-52180.27
12081.60	5540.41
-2104.43	-17208.86
-904.99	-48989.87
-34949.71	-41524.78
13401.69	-32845.20

C

control	db/db
-57.76	-46.80
-68.50	-74.08
31.50	-73.35
20.52	-53.98
-24.22	-63.59
-34.77	-59.23
-31.45	-76.44
-47.66	-74.40
-29.37	-39.81
-32.30	-68.53
-24.94	-116.82
-15.65	-86.34
-34.03	-76.99
19.61	-84.98
-10.79	-82.37
18.67	10.94
-18.66	-61.26
-5.08	-76.54
-48.26	-63.79
28.67	-72.44

Figure S4.

A

Data table contains 9 + 17 columns (aorta samples from control and db/db mice) times 1200 rows (one registered data point every second for 20 minutes). Can be sent upon request from the corresponding author (benyo.zoltan@med.semmelweis-univ.hu).

B

control	db/db
8021.32	1144.86
7372.66	1837.26
998.19	4901.99
12772.96	2216.74
4475.58	5425.83
5650.22	13434.54
4292.62	4754.04
20180.63	22914.27
-130.57	-60348.13
	3783.88
	-1830.97
	28916.15
	31742.24
	33819.68
	-20121.23
	11578.17
	30295.23

C

control	db/db
11.57	4.57
11.12	3.76
-4.77	7.88
14.58	3.57
5.89	6.46
8.32	16.76
6.03	5.51
34.75	31.17
-11.96	-74.46

	-20.21
	-37.84
	43.44
	39.86
	40.14
	-56.65
	25.17
	42.36

Figure S5.

A

Data table contains 5+5+4 columns (aorta samples from control and db/db mice) times 1200 rows (one registered data point every second for 20 minutes). Can be sent upon request from the corresponding author (benyo.zoltan@med.semmelweis-univ.hu).

B

DMSO	MAPP	SKI-II
-27123.79	-40614.84	-52140.9
-29757.79	-56796.94	-32138.0
-49182.09	-17145.67	-48327.5
-47886.42	-49462.75	-65138.6
-50045.13	-49149.72	

C

DMSO	MAPP	SKI-II
-62.33	-64.97	-65.07
-79.35	-82.41	-40.69
-64.09	-62.29	-61.78
-52.05	-70.52	-69.20
-62.56	-80.96	
